# Near vision symptoms and their association with refractive error and binocular vision function among medical sciences students

**DOI:** 10.7717/peerj.21532

**Published:** 2026-06-30

**Authors:** Mohammed M. Alnawmasi, Abdelaziz M. Elmadina, Bandar Alenezi, Sulaiman Aldakhil, Saif Hassan Alrasheed, Nawaf M. Almutairi

**Affiliations:** Department of Optometry, College of Applied Medical Sciences, Qassim University, Buraydah, Qassim, Saudi Arabia

**Keywords:** Symptoms, Quality of life, Binocular vision, Accommodation, Refractive errors, Students

## Abstract

**Background:**

Near-vision-related symptoms are common among students who are often engaged in prolonged near work, such as reading and using digital devices.

**Objective:**

To assess the occurrence of ocular symptoms and their associated visual functions among medical sciences students at Qassim University, Saudi Arabia.

**Methods:**

This cross-sectional study included 58 participants conducted between May and July 2025. Each participant completed a validated Convergence Insufficiency Symptom Survey (CISS) and underwent a comprehensive assessment of refraction, binocular vision and accommodative function.

**Results:**

The findings revealed that 29.3% of participants were classified as symptomatic based on CISS. The most commonly reported symptom was difficulty retaining information after reading (48%), whereas the least reported symptom was the perception of words moving or jumping on the page (7%). Furthermore, uncorrected refractive error significantly influenced symptom severity; ametropic participants reported higher CISS scores (mean ± SD: 18.6 ± 7.5) than emmetropic participants (9.2 ± 5.3, *p* < 0.0001). No statistically significant associations were observed between CISS scores and binocular vision or accommodative function (*p* > 0.05).

**Conclusion:**

The findings highlight that near-vision-related symptoms are prevalent among university students and are significantly influenced by uncorrected refractive errors. Although no significant correlations were identified between symptom severity and binocular vision or accommodative function, these findings underscore the critical role of refractive correction and routine vision screening in academic populations.

## Introduction

Accommodation and non-strabismic binocular vision dysfunction are visual disorders that affect a person’s ability to focus or sustain focus when performing near tasks (Martínez, García Muñoz & Ruiz-Cantero, 2009; Wilkins & Evans, 2024). When binocular vision and accommodative disorders occur, individuals may experience multiple symptoms, including asthenopia, headache, blurred vision, and diplopia (García-Muñoz, Carbonell-Bonete & Cacho-Martínez, 2014). These visual symptoms commonly occur as a consequence of near vision disorders which can affect daily living tasks, including reading or academic achievement (Kovarski et al., 2020; Shin, Park & Park, 2009). A previous study by Alrasheed & Elmadina (2020) investigated the effect of binocular vision problems on childhood academic performance among primary school students (Alrasheed & Elmadina, 2020). They found a significant effect of exophoria at near, positive fusional reserve, and convergence insufficiency on the academic performances of the students.

It has been reported that college students experience a high prevalence of accommodation and non-strabismic binocular vision disorders compared with the general population which could be due to the highest levels of education requiring intensive near work (García-muñoz et al., 2016; Risovic et al., 2008). The growing popularity of electronics, such as computers and smartphones, can be another factor that increases the prevalence of accommodation and non-strabismic binocular vision disorders in this population. Previous studies (Altalhi et al., 2020; García-muñoz et al., 2016; Risovic et al., 2008) on academic populations indicate a high prevalence of near-vision-related symptoms, particularly among students, with 10.8% experiencing accommodative disorders. In Saudi Arabia, the reported prevalence of vision-related symptoms associated with computer vision syndrome in college students is more than 50% which raises a concern regarding diagnosis and intervention to ensure good and comfortable near binocular vision (Altalhi et al., 2020).

Intact visual functions and binocular vision are essential for academic and occupational success since the majority of learning relies on visual skills. In Saudi Arabia, limited research has addressed symptoms related to near-vision tasks within academic populations. Therefore, the present study aimed to assess the occurrence of near-vision-related symptoms among medical sciences students and examine their association with relevant visual and accommodative function.

## Methods

### Study design and setting

This cross-sectional study involved 58 participants from the College of Applied Medical Sciences, Qassim University (Saudi Arabia), conducted between May and July 2025. Each participant completed a validated Convergence Insufficiency Symptom Survey (CISS) and underwent a comprehensive assessment of refraction, binocular vision and accommodative function.

### Sample size

Participants were recruited using a non-probability sampling method from the optometry clinics at Qassim University Medical City. The final sample comprised 58 male and female students, aged between 18 and 26 years.

### Inclusion and exclusion criteria

Participants were considered eligible for inclusion if they were medical sciences students at Qassim University aged 18 to 26 years who signed informed consent, and were able to complete the Arabic version of the CISS-15 and a comprehensive visual assessment. Exclusion criteria included any history of ocular disease, strabismus, prior ocular surgery, systemic/neurologic conditions affecting vision, and medication use known to affect accommodation/binocular vision.

### Ethical approval

Ethical approval for conducting the study was obtained from the Qassim University Health Research Ethics Committee (Approval Number: 24-08-09), and the study was conducted in accordance with the principles of the Declaration of Helsinki. Written informed consent was obtained from participants after explaining the study’s purpose. A consent form explained the study’s objectives, procedures, and timeline. Personal identifiers were removed from the data to ensure confidentiality.

### Data collection procedures

#### Near vision symptoms assessment

Near-vision-related symptoms were assessed using the Arabic version of the CISS (Aljohani & Alnawmasi, 2025). This validated questionnaire has 15 scaled questions and has been widely used to assess near-vision-related symptoms. Each question has five answer options, and each option is scored from 0 to 4, where 0 means “never” and 4 means “always”. Each response’s score was summed to determine the CISS score.

#### Clinical investigations

All participants underwent a comprehensive assessment of basic visual functions, including distance and near visual acuity, contrast sensitivity, refractive errors, and visual field.

1. **Distance Visual Acuity:** Visual acuity was assessed using the FACT program, Freiburg Vision Test (‘FrACT’), an automated procedure for measuring visual acuity (Wesemann, 2002). Participants were seated 3 m from the testing monitor and asked to identify the direction of the Landolt Cs. The participant’s responses were recorded by the examiner on a keyboard as either correct or incorrect. Finally, the results were documented in logMAR.
2. **Near Visual Acuity:** Near visual acuity was measured using the Bailey-Lovie Near Visual Acuity Chart, and the participants were seated 40 cm away from the chart.
3. **Contrast Sensitivity:** Contrast sensitivity was also measured using the FACT program. Participants identified the direction of a Landolt ring (a “tumbling C”) target presented on a monitor (Dennis et al., 2004). Contrast sensitivity (CS) was recorded as Log CS.
4. **Refractive Errors:** Refractive errors were determined using an autorefractor (NIDEK-1, Tokyo, Japan), providing objective measurements of refractive status. Furthermore, Best Corrected Visual Acuity (BCVA) was assessed using trial frames fitted with each participant’s optimal refractive correction. This correction was determined through a full subjective refraction using fogging techniques, whereby plus lenses (or reduced minus power) were introduced to relax accommodation and prevent over-minus findings commonly observed in young adults. Monocular acuity was then measured, testing the right eye first, followed by the left.
5. **Visual Field:** Gross visual field defects were assessed using the confrontation visual field test.

### Binocular vision and accommodation assessment

Participants wore their optimum distance correction during the assessment of binocular vision and accommodation function. All binocular vision and accommodative assessments were conducted by a certified optometrist, following standardized procedures consistent with established optometric practice.

1. Depth perception (stereo acuity) was assessed using VAC FLY Stereo Acuity Test. This test was used to estimate the threshold for local stereopsis by evaluating the participant’s ability to perceive depth in graded circles within boxes. The test measures stereo acuity within a range of 400 to 20 s of arc. In the present study stereo acuity was measured at a distance of 40 centimetres.
2. The Near Point of Convergence (NPC) was measured using the Royal Air Force (RAF) Ruler. The ruler was positioned in front of the participant’s eyes, and they were instructed to focus on the convergence target, which featured a vertical line with a central dot. As the examiner gradually moved the RAF cube closer to the participant’s eyes, the participant was asked to indicate when the target appeared double. The distance at which the target doubled was recorded in centimetres.
3. Phoria: an alternating cover test with prism bar was used to assess the participants’ binocular vision disorders, including latent and manifest strabismus. The amount of prism required to neutralize any detected deviation was recorded in prism dioptre.
4. Positive and negative near-fusional vergence amplitudes were measured using a prism bar. This test measures the amount of prism that can be introduced before the participant experiences sustained blur.
5. Vergence facility was evaluated using a 12 BO/3 BI prism flipper to measure the participant’s ability to rapidly switch vergence without altering accommodation. In the present study, the flipper was alternated by the examiner, and the participant was instructed to read a vertical line of letters from a near chart as soon as the letters appeared clear and single. The test outcome was recorded as the number of cycles per minute.
6. Accommodative Amplitude (AA) was measured both monocularly and binocularly using the RAF ruler with the push-up method and recorded in prism dioptre (D).
7. Accommodative facility was assessed binocularly using a ±2.00 D flipper to evaluate the participant’s ability to adjust accommodation without altering vergence. Using a near chart, the participant was instructed to focus on a letter that was one line larger than their best-corrected visual acuity. The examiner positioned the flipper in front of the participant’s eyes and alternated the lenses each time the participant reported the letter as clear. The result was recorded as the number of cycles per minute, with one cycle defined as the successful clearing of both the plus and minus lenses.

### Statistical analysis

All statistical analyses were performed using SPSS version 24 and GraphPad Prism. Data were expressed as mean ± standard deviation (SD). The normality of data distribution was assessed using the Shapiro–Wilk test. To examine relationships between vision parameters and CISS score, Spearman rho (p) Correlation coefficients (p) analysis was used because these data were not normally distributed. Comparisons between the Emmetropic and Ametropic groups were conducted using the independent samples *t*-test.

## Results

A total of 58 participants (43 males and 15 females) with a mean age of 22.8 ± 2.4 years completed the study. Among them, 20 (34.5%) reported wearing spectacles. Using a threshold of 21 or higher to define symptomatic individuals (Rouse et al., 2004), 29.3% of the subjects were classified as symptomatic, while 70.7% were asymptomatic.

Table 1 presents the percentage distribution of responses to each CISS question for all subjects. The most frequently reported symptom among the subjects was trouble remembering what had been read, with 48% experiencing it sometimes, fairly often, or always. The least common symptom was perceiving words as moving, jumping, or floating across the page while reading or doing close work (7%).

The total CISS score did not show a significant correlation with any of the binocular vision and accommodation functions, as shown in Table 2. However, visual acuity showed a statistically significant inverse correlation with the CISS score (*r* = −0.375, *p* = 0.004), indicating that symptoms increased as visual acuity worsened. Therefore, further analysis was conducted to assess whether emmetropic participants would have a similar CISS score as ametropic participants for both the CISS composite score and individual questions.

**Table 1:** Percentage of answers for each question of the CISS-related symptoms.

**Q#**	**Question**	**Never**	**Infrequently (not very often)**	**Sometimes**	**Fairly often**	**Always**
1.	Do your eyes feel tired when reading or doing close work?	31%	24%	34%	7%	3%
2.	Do your eyes feel uncomfortable when reading or doing close work?	41%	31%	19%	5%	3%
3.	Do you have headaches when reading or doing close work?	50%	26%	19%	5%	0%
4.	Do you feel sleepy when reading or doing close work?	38%	22%	28%	10%	2%
5.	Do you lose concentration when reading or doing close work?	33%	26%	31%	7%	3%
6.	Do you have trouble remembering what you have read?	33%	19%	24%	17%	7%
7.	Do you have double vision when reading or doing close work?	69%	16%	9%	5%	2%
8.	Do you see the words move, jump, swim or appear to float on the page when reading or doing close work?	81%	12%	5%	0%	2%
9.	Do you feel like you read slowly?	50%	24%	17%	5%	3%
10.	Do your eyes ever hurt when reading or doing close work?	52%	24%	19%	3%	2%
11.	Do your eyes ever feel sore when reading or doing close work?	33%	38%	21%	5%	3%
12.	Do you feel a “pulling” feeling around your eyes when reading or doing close work?	62%	21%	12%	2%	3%
13.	Do you notice the words blurring or coming in and out of focus when reading or doing close work?	55%	26%	17%	0%	2%
14.	Do you lose your place while reading or doing close work?	52%	31%	9%	7%	2%
15.	Do you have to re-read the same line of words when reading?	33%	33%	14%	12%	9%

**Table 2:** Spearman rho (P) Correlation coefficients (p) analysis of different parameters and their *p*-values.

**Variable**	**Correlation** **analysis**	**VA OU**	**CS OU**	**Stereo acuity**	**CISS score**
Age	*p*	−0.118	**0**.**269**	−0.096	−0.021
	*P* value	0.378	**0**.**043**	0.504	0.873
VA OU	*p*	1.000	**0**.**593**	0.043	**−0**.**375**
	*P* value	**“<0**.**001”**	**<0**.**001**	0.766	**0**.**004**
CS OU	*p*	**0**.**593**	1.000	−0.016	0.146
	*P* value	**“<0**.**001”**	.	0.914	0.279
NPC (Break)	*p*	0.164	−0.009	0.040	0.150
	*P* value	0.232	0.949	0.785	0.274
Amplitude of accommodation	*p*	0.071	**−0**.**333**	−0.133	−0.080
	*P* value	0.597	**0**.**011**	0.352	0.550
Accommodative facility	*p*	0.239	−0.258	**−0**.**486**	0.156
	*P* value	0.074	0.054	**<0**.**001**	0.246
Vergence facility	*p*	0.123	−0.035	**−0**.**489**	0.152
	*P* value	0.417	0.820	**0**.**001**	0.313
Stereo acuity	*p*	0.043	−0.016	1.000	0.049
	*P* value	0.766	0.914	.	0.731
CISS	*p*	**−0**.**375**	0.146	0.049	1.000
	Sig. (2-tailed)	**0**.**004**	0.279	0.731	.
PFV	*p*	−0.170	**0**.**512**	−0.085	−0.056
	*P* value	0.224	**<0**.**001**	0.570	0.691
NFV	*p*	−0.152	0.183	**−0**.**345**	0.074
	Sig. (2-tailed)	0.266	0.185	**0**.**015**	0.591

**Notes.**

VA Visual acuity NPC Near point of convergence CS contrast sensitivity PFV Positive fusional vergence NFV Negative fusional vergence

Both male and female participants showed a comparable CISS score (t = −0.51 (1,56), *p* = 0.66). The individual question was compared between male and female participants, and the results showed no significant difference.

### Symptom score and refractive status

A separate analysis was conducted to investigate whether refractive status influences the symptom score. Participants were divided based on their reactive status of either emmetropic participants who have a spherical equivalent of less than ±0.50 D or ametropic participants who have a spherical equivalent of ±0.50 D or more. This was consistent with definitions commonly used in epidemiological studies of refractive error (Galvis et al., 2021). Although this threshold may be considered low in term of clinical prescribing criteria, it was selected to include even mild refractive errors that may be functionally significant in young adults engaged in sustained near work.

**Figure 1:**
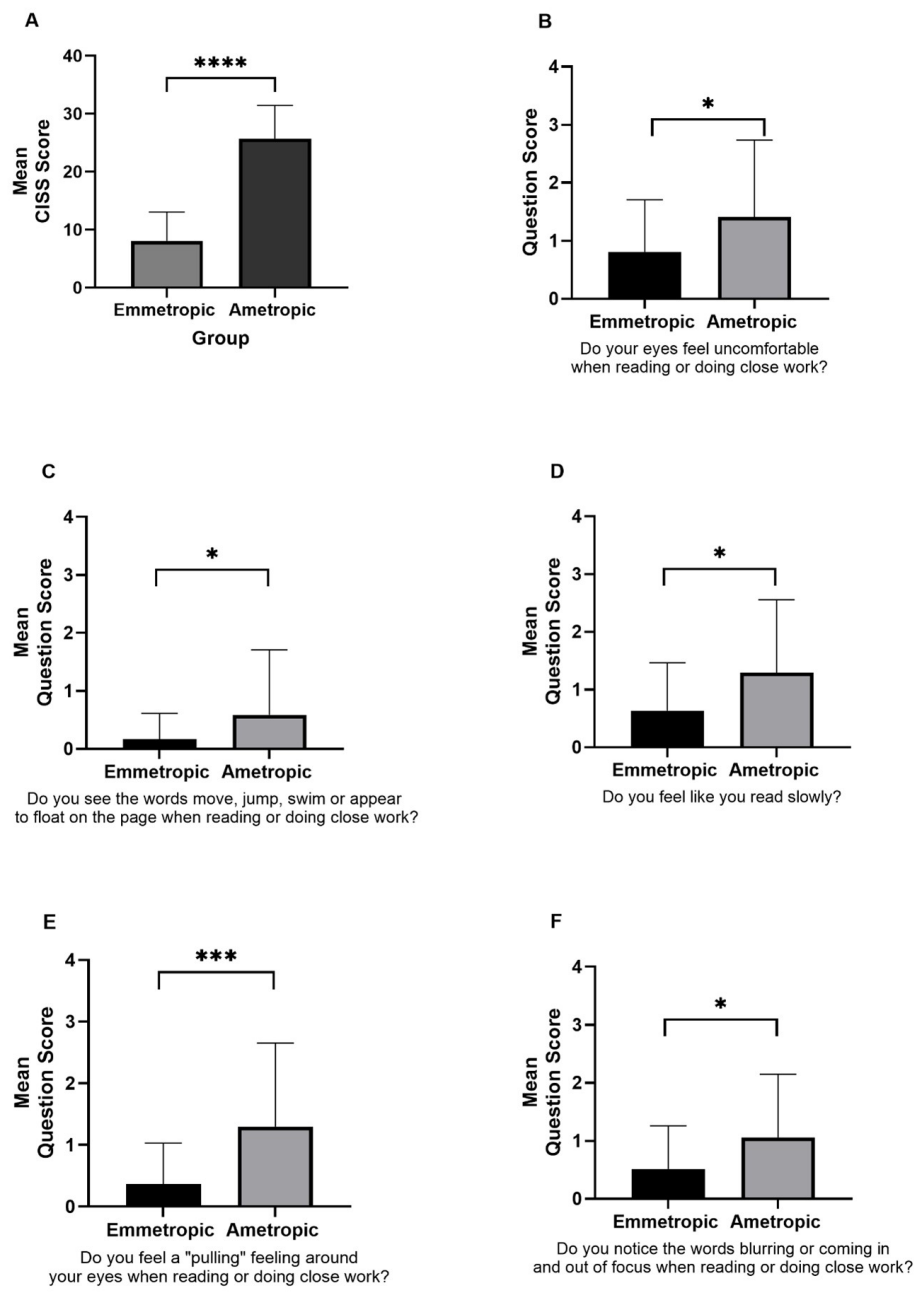
Mean CISS score and selected individual questions between emmetropic and ametropic participants.

The results indicate that ametropic participants experience significantly higher visual discomfort and reading difficulties compared to their emmetropic counterparts. Figure 1A illustrates the mean CISS score for both groups, demonstrating a statistically significant difference (t(56) = 11.76, *p* < 0.0001, *η*^2^ = 0.71), where ametropic participants exhibit considerably higher symptom scores.

Figure 1B through 1F show the specific visual symptoms that were significantly higher in the ametropic group. In Fig. 1B, ametropic participants report significantly higher discomfort during close work (t(56) = 2.02, *p* = 0.04, *η*^2^ = 0.1). Figure 1C shows that ametropes are more likely to perceive words moving, jumping, or floating on the page (t(56) = 2.05, *p* = 0.04, *η*^2^ = 0.07). Figure 1D highlights that ametropic participants feel they read more slowly than emmetropic participants (t(56) = 2.35, *p* = 0.02, *η*^2^ = 0.1). In Fig. 1E, the feeling of “pulling” around the eyes is reported significantly more by ametropic participants (t(56) = 3.51, *p* < 0.001, *η*^2^ = 0.1). Finally, Fig. 1F demonstrates that ametropic participants are more likely to notice blurring or fluctuations in focus during near work (t(56) = 2.21, *p* = 0.03, *η*^2^ = 0.08). Overall, these results indicate that ametropia is associated with significant visual discomfort and difficulties in near-vision tasks.

## Discussion

The current study aimed to evaluate near vision-related symptoms and their associated visual functions among medical sciences students in Qassim University, Saudi Arabia. The results show a high occurrence of near-vision symptoms in this population, where 29.3% of participants were classified as symptomatic according to the CISS. Comparable findings have been reported in previous studies, which identified a high prevalence of accommodative and non-strabismic binocular vision disorders among academic populations, particularly those engaged in prolonged near-vision tasks (Alrasheed & Elmadina, 2020; Shin, Park & Park, 2009).

A key finding of this study is that the most commonly reported symptom was difficulty remembering what was read, in which 48% of participants experienced varying frequencies. This suggests that near-vision difficulties may negatively impact cognitive processing and comprehension, potentially affecting academic performance. Similar results have been observed in previous studies, where poor binocular vision was associated with decreased reading efficiency and academic performance (García-Muñoz, Carbonell-Bonete & Cacho-Martínez, 2014). In contrast, the least frequently reported symptom was perceiving words as moving or jumping while reading, suggesting that visual distortions are less common than symptoms related to ocular discomfort and concentration. Furthermore, the memory symptoms may reflect cognitive burden secondary to inefficient visual processing rather than purely due to refractive problems. Beyond refractive correction, several optometric and ergonomic strategies might support these students, including improving near working distance, enhancing lighting condition and posture, and encouraging regular visual breaks such as 20/20/20 rule to reduce accommodative and vergence fatigue. Additionally, addressing binocular vision disorders through targeted vision therapy particularly activities that enhance accommodative and vergence strength may help reduce the cognitive burden associated with sustained near tasks. Such interventions can improve overall reading comfort and may indirectly support better retention of information and relief the memory symptoms.

Interestingly, the total CISS score did not show a significant correlation with binocular vision functions, suggesting that symptoms may not always be directly linked to the objective binocular vision measures. This finding is consistent with previous research, which has reported that self-reported visual symptoms often do not correlate strongly with clinical measurements of binocular vision (Bade et al., 2013; Horwood, Toor & Riddell, 2014).

Further analysis indicated that uncorrected refractive error was significantly associated with higher CISS scores. Specifically, ametropic participants reported significantly more symptoms compared to emmetropic participants. This finding emphasizes the importance of refractive errors as a contributing factor to near-vision discomfort, supporting previous studies that have demonstrated that uncorrected refractive error might be a confounding factor when considering visual symptomatology (Cacho-Martínez et al., 2015). Moreover, the specific ametropia included in this study included myopia, hyperopia, and astigmatism, these refractive errors differ in their likelihood of producing visual symptoms, with uncorrected hyperopia and astigmatism generally causing more near-vision discomfort than simple myopia. The higher symptom scores in ametropic participants were particularly pronounced for questions related to eye soreness, pulling sensations around the eyes, and blurred vision. This highlights the need for regular vision screenings among students to identify and correction of refractive errors. Furthermore, in the current study, visual acuity demonstrated a statistically significant inverse correlation with CISS scores (*r* = −0.375, *p* = 0.004), indicating that symptom severity increased as VA declined. This relationship may reflect the more immediate and noticeable impact of reduced visual clarity on near-vision comfort. In contrast, binocular and accommodative anomalies may remain subclinical in young adults due to their greater adaptive capacity, such as accommodation amplitude and fusional reserve, which could explain the absence of significant correlations with symptom scores.

Although the mean CISS score in the ametropic group did not exceed the clinical cutoff value of 21, ametropic participants demonstrated significantly higher symptom scores compared to emmetropic participants. This indicates an increased burden of visual discomfort that may be considered subclinical but functionally relevant, particularly during prolonged near tasks. It is important to note that the CISS in this study reflects symptoms under habitual visual conditions and given that a substantial proportion of participants did not routinely wear refractive corrections, the findings likely represent the impact of uncorrected ametropia in daily life. Therefore, even small increases in symptom scores may have practical implications for visual comfort and academic performance.

In the present study, an important factor in interpreting the association between refractive errors and symptom severity is the high prevalence of habitually uncorrected ametropia among the participants. While all students underwent clinical assessment using their optimum distance correction, only 34.5% reported wearing spectacles in daily life. This suggests that many students in the ametropic group were commonly functioning without adequate refractive correction, which likely contributed to the increased symptom severity shown in this study. Therefore, the findings may reflect the impact of uncorrected refractive error in daily visual tasks rather than a fully corrected refractive status.

Accommodation and vergence dysfunctions were also evaluated in this study. Although the binocular vision and accommodation parameters did not show a significant correlation with symptoms, accommodative facility and vergence facility were negatively correlated with stereo acuity. These findings suggest that participants with reduced accommodative and vergence flexibility may experience difficulties with depth perception, potentially contributing to visual symptoms such as blurred vision, difficulty with depth perception, and visual fatigue during sustained near tasks such as reading or computer work (Piñero, 2020).

Regarding the findings of the present study, it is important to recognize that environmental and behavioral factors may also influence near-vision symptoms in medical students. Even though the current study did not record external variables such as average daily digital device use, duration of near-work activities, or study-environment lighting conditions, these factors are known to contribute to visual discomfort and may affect CISS scores. The intensive near-work demands typical of medical training could therefore act as potential confounders in the observed associations. Future studies should include comprehensive assessments of digital device usage patterns and environmental conditions to better control for their influence on symptomatology and to provide a more comprehensive understanding of the determinants of near-vision–related symptoms in this population.

The present study found that accommodative and vergence facility were negatively and statistically significantly correlated with stereo acuity. The negative correlation between accommodative and vergence facility and stereo acuity may suggest that reduced oculomotor flexibility may compromise the stability of binocular fusion during near tasks. Accommodative and vergence facility commonly represent the dynamic reaction of the visual system; when this flexibility is reduced, the ability to sustain precise alignment and focus becomes less efficient. Consequently, stereo acuity may deteriorate due to intermittent fusion instability. While depth perception is not directly required for reading, reduced stereo acuity may reflect a fundamental binocular vision stress that can manifest during prolonged near work. Thus, students with limited accommodative or vergence facility may therefore experience increased visual effort, reduced reading comfort, and potential performance decline during sustained reading tasks. This relationship underscores the importance of assessing accommodation and vergence facility in people reporting near-vision symptoms, as they may serve as early indicators of functional binocular dysfunction.

## Limitations and Future Directions

Despite the valuable insights provided by this study, some limitations might limit the generalization of the findings. First, participants were recruited using non-probability sampling from optometry clinics, which introduces the potential for selection bias and may overestimate symptom prevalence, thereby limiting the external validity of the findings. Second, the relatively small sample size (*N* = 58) restricts the generalizability of the results to broader student populations and may reduce the statistical power to detect meaningful associations. These factors should be considered when interpreting the study outcomes, and future research with larger, more representative samples and rigorous sampling methods is recommended to strengthen the evidence base. A more balanced cohort is needed to confirm these findings and to more accurately examine potential gender-related differences in near-vision symptoms, as the unequal gender distribution in the present study limits subgroup comparisons. Additionally, future studies with larger sample sizes should investigate the differential effects of specific refractive error types on symptomatology. Moreover, the study relied on self-reported symptoms using the CISS questionnaire, which, although validated, may introduce subjective bias. Future research may consider comparing visual symptoms across different academic fields to identify whether certain groups are at higher risk for near-vision disorders. Finally, longitudinal studies tracking changes in symptoms over time, particularly in response to interventions such as vision therapy or refractive corrections, would provide valuable insights into effective management strategies.

## Conclusion

The findings indicate that near-vision–related symptoms are common among university students and are significantly associated with uncorrected refractive errors. Although no significant correlations were identified between symptom severity and binocular vision or accommodative function, these findings underscore the critical role of refractive correction and routine vision screening in academic populations. These findings emphasize the importance of comprehensive vision assessments for students engaged in prolonged near work and support the need for targeted interventions to improve visual comfort and academic performance.

##  Supplemental Information

10.7717/peerj.21532/supp-1 Supplemental Information 1 Raw Data

10.7717/peerj.21532/supp-2 Supplemental Information 2 STROBE Checklist

## Additional Information and Declarations

### Data Availability

The following information was supplied regarding data availability: The raw data is available in the Supplemental File.
